# The Adverse Cardiovascular Effects and Cardiotoxicity of Kratom (*Mitragyna speciosa* Korth.): A Comprehensive Review

**DOI:** 10.3389/fphar.2021.726003

**Published:** 2021-09-27

**Authors:** Mohammad Farris Iman Leong Bin Abdullah, Darshan Singh

**Affiliations:** ^1^ Lifestyle Science Cluster, Advanced Medical and Dental Institute, Universiti Sains Malaysia, Kepala Batas, Malaysia; ^2^ Centre for Drug Research, Universiti Sains Malaysia, Gelugor, Malaysia

**Keywords:** cardiovascular adverse effects, cardiotoxicity, kratom related mortality, kratom use, QTc interval, literature review

## Abstract

**Background:** Kratom or *Mitragyna speciosa* (Korth.) has received overwhelming attention recently due to its alleged pain-relieving effects. Despite its potential therapeutic value, kratom use has been linked to many occurrences of multiorgan toxicity and cardiotoxicity. Accordingly, the current narrative review aimed to provide a detailed account of kratom’s adverse cardiovascular effects and cardiotoxicity risk, based on *in vitro* studies, poison center reports, coroner and autopsy reports, clinical case reports, and clinical studies.

**Methods:** An electronic search was conducted to identify all research articles published in English from 1950 to 2021 using the major research databases, such as Google Scholar, Web of Science, PubMed, Scopus, Mendeley, EMBASE, Cochrane Library, and Medline. We then analyzed the literature’s discussion of adverse cardiovascular effects, toxicity, and mortality related to kratom use.

**Results:** Our findings revealed that, although *in vitro* studies have found kratom preparations’ most abundant alkaloid—*mitragynine*—to cause a prolonged QTc interval and an increased risk of torsades de pointes, a clinical study examining humans’ regular consumption of kratom did not report such a risk. However, this latter study did show that regular kratom use could induce an increased QTc interval in a dose-dependent manner. A few case reports also highlighted that kratom consumption is associated with ventricular arrhythmia and cardiopulmonary arrest, but this association could have ensued when kratom was co-administered with another substance. Similarly, analyses of national poison data showed that kratom’s most common adverse acute cardiovascular effects include tachycardia and hypertension. Meanwhile, coroner and autopsy reports indicated that kratom’s cardiovascular sequelae encompass coronary atherosclerosis, myocardial infarction, hypertensive cardiovascular disease, left ventricular hypertrophy, cardiac arrhythmia, cardiomegaly, cardiomyopathy, focal band necrosis in the myocardium, and myocarditis. Given the available data, we deduced that all cardiac eventualities reported in the literature could have been compounded by polysubstance use and unresolved underlying medical illnesses.

**Conclusion:** Although kratom use has been associated with death and cardiotoxicity, especially at higher doses and when associated with other psychoactive drugs, the dearth of data and methodological limitations reported in existing studies do not allow a definitive conclusion, and further studies are still necessary to address this issue.

## Introduction

*Mitragyna speciosa* (Korth.) or kratom is an indigenous medicinal plant in the Rubiaceae family that can be widely found in its natural habitat of Southeast Asia, particularly in Thailand, Malaysia, and Indonesia. Its leaves are dark green in color and oval in shape, and they have been traditionally consumed by rural inhabitants of Southern Thailand and Northern Peninsular Malaysia for centuries. This traditional use has relied on kratom to symptomatically relieve muscle pain, cough, fever, and diabetes mellitus. Moreover, the plant has also been traditionally used in these areas as an aphrodisiac. For the past decade, kratom has become popular in the West (the United States and Europe), where it is mainly used for its broad antidepressant, anxiolytic, and analgesic properties as a safe substitute for prescription drugs and for illicit opioid or heroin use. Kratom has also been used in the West for its dose-dependent stimulant and sedative-like psychoactive effects. Unlike in Southeast Asia, where fresh kratom leaves are used to produce kratom decoctions (kratom tea or juice), kratom in the West is largely ingested as a dried leaf powder (Hassan et al., 2013; Singh et al., 2016; Leong Bin Abdullah et al., 2020; Domnic et al., 2021).

A wide variety of kratom products are currently sold online in the form of resin, dried leaves, or raw leaf extracts. However, these products’ psychoactive content is unknown. Following reports about the addictive potential and various possible toxicities associated with kratom use, several countries have categorized kratom as a controlled substance. In Malaysia, mitragynine (the most abundant psychoactive alkaloid of kratom extracts) has been included in the Dangerous Poison Act 1953 since 2003. Although the planting of kratom trees is not considered an offense in Malaysia, the trafficking and possession of kratom leaves are illegal, and people convicted of these criminal acts could be penalized with prison sentences of up to 4 years, a maximum fine of 10,000 Malaysian Ringgit, or both of these punishments (Vicknasingam et al., 2010). In Thailand, kratom had previously been placed under Schedule 5 of the Thai Narcotic Act. Recently, however, kratom was removed from this schedule after an amendment to the act was passed. However, the cultivation of kratom products remains restricted under the country’s new law (Vicknasingam et al., 2010; Bangkok Post, 2021). In Indonesia, the cultivation of kratom is permitted for commercial purposes, and kratom is exported to other countries in Asia, Europe, and America. However, under a new regulation of the Indonesian National Narcotics Agency (BNN) that will take effect in 2022, kratom will be an illegal substance.

In the international context, kratom is classified as a controlled substance in countries such as Myanmar, Australia, Sweden, Denmark, Poland, Latvia, Lithuania, and Romania. In the United Kingdom, the export, import, and sale of kratom are prohibited under the Psychoactive Substances Act. Although kratom is not a controlled substance in the United States, it has been scrutinized by the US Drug Enforcement Administration (Hassan et al., 2013; Eastlack et al., 2020). However, in 2018, the US Food and Drug Administration (FDA) issued a warning against the therapeutic use of kratom, claiming that the substance is an opioid with harmful effects that could cause abuse, dependence, and even death (Gershman et al., 2019). Due to kratom’s potential to induce toxicity, it has been placed on the controlled substance lists of several US states—such as Alabama, Arkansas, Indiana, Tennessee, Wisconsin, Rhode Island, and Vermont (Eastlack et al., 2020).

Although more than 40 chemical compounds have been isolated from kratom leaves, only four alkaloids are known to be pharmacologically active: mitragynine, 7-hydroxymitragynine (7-HMG), corynantheidine, and speciociliatine (Chear et al., 2021). Among these compounds, mitragynine and its metabolite 7-HMG have been researched the most. Mitragynine is the most abundant alkaloid, contributing to 66% of kratom’s total alkaloid content. Meanwhile, kratom preparations’ 7-HMG content is much lower (only 0.02% of their total alkaloid content) (Takayama, 2004; Kruegel and Grundmann, 2018). Mitragynine and 7-HMG mainly bind to opioid receptors. Notably, mitragynine, and 7-HMG’s affinities for the opioid receptor subtypes differ. Mitragynine has been reported to have a higher affinity for the *µ* and *δ* receptors while 7-HMG has exhibited a higher affinity for the *µ* and *κ* receptors. Unlike morphine, which is a *µ* and *δ* receptor agonist, mitragynine, and 7-HMG may be partial *µ* receptor agonists and *δ* receptor antagonists (Kruegel et al., 2016). Another notable difference is that mitragynine and 7-HMG are G-protein-coupled and not involved in the activation of β-arrestin signaling, unlike morphine. Therefore, kratom has been reported to induce less opioid-like adverse effects or toxicity than morphine, which has been shown to cause respiratory depression, constipation, and sedation (Raehal et al., 2011; Wisler et al., 2014).

Despite an expectation that kratom could induce less adverse or toxic effects than opioids, the toxicity related to kratom use has been reported cumulatively, and it involves many organ systems: 1) kratom-induced liver injury, such as hepatitis, raised liver enzymes, hepatomegaly, acute liver failure, intrahepatic cholestasis, and severe liver injury with jaundice (Dorman et al., 2015; Griffiths et al., 2018; Waters et al., 2018; Fernandes et al., 2019; Osborne et al., 2019; Ahmad et al., 2021); 2) endocrinal defects, such as hypothyroidism (Sheleg and Collins, 2011); 3) neurological defects, such as seizures, coma, and memory impairment (Nelsen et al., 2010; Tatum et al., 2018; Singh et al., 2019); 4) respiratory defects, such as pulmonary edema and congestion (McIntyre et al., 2015); 5) renal injury, such as acute renal failure (Sangani et al., 2021); 6) muscular injury, such as rhabdomyolysis and compartment syndrome (Sangani et al., 2021); and neonatal abstinence syndrome among infants born to mothers who used kratom during pregnancy (Eldridge et al., 2018; Mitra and Virani, 2018). Evidence of possible cardiotoxicity due to kratom exposure was first documented in an *in vitro* study of human-induced pluripotent stem-cell-derived cardiomyocytes (hiPSC-CMs); this study reported that mitragynine and its analogs increased the risk of prolonged QTc interval and torsades de pointes (Lu et al., 2014). To the best of our knowledge, to date, comprehensive studies detailing the adverse cardiovascular effects and cardiotoxicity of kratom use have been lacking. Therefore, we conducted a comprehensive literature review incorporating *in vitro* studies, poison center reports, coroner and autopsy reports, clinical case reports, and clinical studies to provide a detailed view of this subject.

## Materials and Methods

An electronic search was conducted on literature published from 1950 to 2021. This search was conducted independently by this review’s two authors (MFILA and DS) using the major research databases, such as Google Scholar, Web of Science, PubMed, Scopus, Mendeley, EMBASE, Cochrane Library, and Medline. The search terms and keywords used included “kratom,” “*Mitragyna speciosa*,” “*Mitragyna speciosa* Korth,” “*M.speciosa* adverse effects,” “kratom risks and benefits,” “*M.speciosa* toxicity,” “kratom cardiotoxicity,” “*in vitro* study of kratom cardiotoxicity,” “animal study of kratom cardiotoxicity,” and “kratom-related death.” An initial search yielded a total of 170 articles From these initially identified articles, our selection was refined according to our search criteria, which determined that literature was eligible for review if it was: 1) published in an English-language peer-reviewed journal, including in-press articles, 2) a research article, case report, or case series, and 3) related to the adverse cardiovascular effects and cardiotoxicity of kratom use. Literature was excluded from this review if it was: 1) published in non-English-language journals (because the current authors could not access an expert who could interpret non-English-language studies’ content and findings), 2) a systematic review, narrative review, unpublished article, or thesis, 3) described *Mitragyna tubulosa*, *Mitragyna parvifolia*, *Mitragyna rotundifolia*, *Mitragyna hirsuta*, *Mitragyna savanica*, *Mitragyna inermis*, *Mitragyna africanus*, *Mitragyna Rubro stipulata*, or *Mitragyna ciliata*, or 4) addressed aspects of kratom-related toxicities other than cardiotoxicity. Therefore, after thorough analysis, only 11 identified articles were ultimately selected for inclusion in this review. A summary of these selected articles is presented in Table 1. The selected studies in Table 1 are presented according to the hierarchy of evidence proposed by Sayre et al. (2017) from the lowest evidence level to the highest evidence level.

**TABLE 1:** Summary of reviewed literature.

Author (year)	Study design, sampling, and sample size	Sample size calculation (Yes/No)	Objectives	Outcome measures	Findings	Limitations
Lu et al. (2014)	*In vitro* study with hiPSC-CMs	—	To investigate the cardiotoxicity of mitragynine and its analogs by studying their effects on hERG and APD	(1) IKr	(1) Mitragynine, paynantheine, speciogynine, and speciociliatine suppressed IKr in hiPSC-CMs in a dose-dependent manner	(1) hiPSC-CMs contain different subtypes of cardiomyocytes
						(2) hiPSC-CMs are immature and embryonic-like compared to adult cardiomyocytes
				(2) ICa,L	(2) Mitragynine significantly prolonged APD, which induced prolonged QTc and with the potential of causing torsades de pointes	
				(3) APD	(3) Mitragynine did not cause synthesis or trafficking defects of hERG	
Tay et al. (2019)	*In vitro* study with hERG1a/1b-transfected HEK293 cells	—	To determine the mechanisms of mitragynine-induced inhibition on hERG1a/1b current	The effects of mitragynine on: (1) hERG1a/1b expression	(1) Mitragynine inhibited the cardiac IKr current in a concentration-dependent manner	(1) Used transfected HEK293 cells instead of cardiomyocytes
				(2) hERG1-cytosolic chaperones’ interaction		
					(2) Mitragynine had no inhibitory or induction effects on the mRNA expression of hERG1a and hERG1b	
					(3) Mitragynine reduced fully glycosylated (fg) hERG1a but upregulated both core-glycosylated (cg) expression and hERG1a-Hsp90 complexes	
					(4) In conclusion, mitragynine may impair hERG1a trafficking by preventing proper hERG1a channel protein folding through the plasma membrane of transfected HEK293 cells	
Aggarwal et al. (2018)	Case report	—	—	—	A 26-year-old man: (a) History: presented with cardiorespiratory arrest after ingesting an unknown quantity of kratom 24 h previously; no prior medical illness or regularly prescribed medication	(1) The patient consumed a standard dose of codeine
					(b) Clinical findings: cardiorespiratory arrest with ventricular arrhythmia	(2) Serum mitragynine and 7-HMG were not measured
					(c) Investigations	
					(i) Urine toxicology: the presence of codeine (of which the patient had taken a standard dose just prior to admission)	
					(ii) Other findings: imminent cerebral herniation in CT brain scan	
					(d) Outcome: the patient died 12 h after initial ROSC	
Abdullah et al. (2019)	Case report	—	—	—	A 35-year-old man: (a) History: presented with cardiorespiratory arrest and a history of taking kratom in powdered form as a tea numerous times daily; history of polysubstance abuse; used kratom as self-prescribed medication for opioid dependence	(1) The kratom powder that the patient consumed could have been adulterated
					(b) Clinical findings: cardiovascular, gastrointestinal, and respiratory examinations were otherwise unremarkable; a neurological examination revealed only evidence of cardiorespiratory arrest	(2) Serum mitragynine and 7-HMG were not assessed
					(c) Investigations	
					(i) Arterial blood gas: respiratory acidosis, liver function test: liver impairment	
					(ii) Cardiac enzyme analysis: high creatinine kinase (4,000 U/L) and troponin I (0.37 μ/L)	
					(iii) ECG findings were normal and an echocardiogram only indicated a recent cardiac arrest	
					(iv) Other investigations were unremarkable and a urine drug screen upon admission was negative for any drugs	
					(d) Outcome: patient survived and recovered from opioid withdrawal symptoms 8 days after admission	
ELJack et al. (2020)	Case report	—	—	—	A 24-year-old man: (a) History: presented with cardiorespiratory arrest with a history of continually using illicit substances, particularly kratom, but had abstained from opioid use for approximately 1 year; history of polysubstance abuse but no history of medical illness prior to the incident	(1) Serum mitragynine and 7-HMG were not assessed
					(b) Clinical findings: physical examination revealed unremarkable findings	(2) Likely co-exposure of kratom and other substances
					(c) Investigations	
					(i) Cardiovascular investigation: ventricular fibrillation (polymorphic ventricular tachycardia) and incomplete right bundle branch block in ECG	
					(ii) Transthoracic echocardiography: normal	
					(iii) Other investigation: indicative of tissue and organ hypoperfusion due to cardiac arrest	
					(iv) Serum and urine toxicology screening: no evidence of any illicit drug use or medication overdose	
					(d) Outcome: Patient fully recovered and was extubated 2 days after his hospital presentation	
Sheikh et al. (2021)	Case report	—	—	—	A 44-year-old man: (a) History: presented with cardiorespiratory arrest and a history of consuming kratom daily as an energy supplement, co-administered with an energy drink; otherwise, no history of underlying medical illnesses	(1) No assessment of serum mitragynine and 7-HMG
					(b) Clinical findings: unremarkable	(2) Co-exposure of kratom and other substances
					(c) Investigations	
					(i) Cardiovascular investigation: multiple episodes of ventricular fibrillation and later prolonged QT interval and intraventricular conduction block in ECG	
					(ii) Chest x-ray: pulmonary vascular congestion	
					(iii) Emergency cardiac catheterization, ECG (no left ventricular abnormalities), cardiac MRI, and serum troponin were all normal	
					(d) Outcome: Patient fully recovered	
Anwar et al. (2016)	(1) Retrospective survey	—	Not mentioned	(1) Single exposure versus multiple exposures	Cardiovascular finding: (1) Common adverse cardiovascular effects were tachycardia (25%) and hypertension (11.7%)	(1) Unverified reports
	(2) Sample size: 660 reports of kratom exposure			(2) Common substances co-administered with kratom	Other findings: (1) Isolated kratom exposure was reported in 64.8% of cases	(2) Unknown health backgrounds in cases
				(3) Symptoms and signs of kratom exposure	(2) Common co-administered substances included ethanol, other botanicals, benzodiazepines, narcotics, and acetaminophen	(3) Serum mitragynine and 7-HMG levels not available
				(4) Factors associated with outcomes’ severity	(3) Multiple exposures (kratom co-administration with other substances) increased the risk of a severe outcome compared to a single exposure	
Post et al. (2019)	(1) Retrospective survey	—	To analyze reports of kratom exposure to the US NPDS from 2011 to 2017	(1) Single exposure vs. multiple exposures by age group	Cardiovascular finding: (1) Adverse cardiovascular effects: tachycardia (21.4%), hypertension (10.1%), conduction defects (2.8%), chest pain (including non-cardiac pain; 2.6%), hypotension (1.8%), bradycardia (1.2%), and cardiac arrest (0.4%)	(1) Unverified reports
	(2) Sample size: 1,807 reports of kratom exposure			(2) Trend of kratom exposure from 2011 to 2017	Other findings: (1) 65% of cases reported involved only kratom exposure	(2) Unknown health backgrounds in cases
				(3) Clinical features and medical outcomes associated with kratom exposure	(2) 11 kratom-related deaths were reported with only two cases associated with isolated kratom exposure	(3) Serum mitragynine and 7-HMG levels not available
Davidson et al. (2021)	(1) Retrospective survey	—	To analyze reports of kratom exposure with abuse potential to the US NPDS and Thai RPC from 2011 to 2017	(1) Characteristics of kratom exposure	Cardiovascular findings: (1) Adverse cardiovascular effects and outcomes: tachycardia (30.4%) and hypertension (12.4%)	(1) Unverified reports
	(2) Sample size: 928 reports of kratom exposure			(2) Trend of kratom exposure from 2011 to 2017	Other findings: (1) Thailand registered a higher prevalence of co-exposure of kratom with other substances than the United States	(2) Unknown health backgrounds in cases
				(3) Single exposure vs. multiple exposures	(2) The United States reported more co-ingestion with other sedatives than Thailand	(3) Serum mitragynine and 7-HMG levels not available
				(4) Prevalence of co-ingested substances	(3) Five out of six reported deaths were associated with the co-ingestion of kratom and other substances	(4) Kratom dosing and formulation not available
				(5) Common clinical effects of kratom exposure		
				(6) Factors associated with death and ICU admission		
Corkery et al. (2019)	(1) Retrospective survey	—	To examine the nature of death reportedly associated with kratom exposure across the United Kingdom, United States, Europe, and Thailand until 2019	(1) The main characteristics of deaths associated with kratom use	Cardiovascular finding: (1) Frequency of cardiovascular findings in deaths solely attributed to kratom: *n* = 9, 5.8%	(1) Questionable quality of some data sources
	(2) Sample size: 156 kratom-related mortality cases			(2) Serum mitragynine and 7-HMG levels among patients who had died	(2) Frequency of cardiovascular findings in deaths attributed to kratom combined with other substances: *n* = 18, 11.5%	
				(3) Frequency of kratom exposure only and co-exposure	(3) Frequency of cardiovascular findings in deaths in which kratom’s role was unclear: *n* = 5, 3.2%	
				(4) Main causes of death and autopsy reports associated with kratom exposure only and co-exposure	Other findings: (1) Exposure to kratom alone constitutes 23% of death cases while polysubstance use was reported in 87% of death cases	
					(2) Serum mitragynine levels in mortality cases were as follows	
					(a) Death solely attributed to kratom (mean = 0.398 mg/L, range 0.0035–0.890 mg/L; *n* = 3)	
					(b) Death attributed to kratom combined with other substances (mean = 0.8903 mg/L, range 0.00089–16.000 mg/L; *n* = 62)	
Leong Abdullah et al. (2021)	(1) Analytical, cross-sectional study	Yes	To investigate the prevalence of ECG abnormalities generally and QTc intervals particularly among regular kratom users versus non-kratom-using control participants	(1) Kratom use characteristics	(1) Kratom users (8%) had significantly higher odds of sinus tachycardia than control participants (1%); no significant difference was found in other ECG abnormalities	(1) Cross-sectional design
	(2) Snowball sampling			(2) Resting ECG	(2) An age during one’s first experience of kratom consumption of >18 years old, a consumption duration of > 6 years, and daily kratom juice consumption quantity of one to four glasses significantly increased one’s odds of a borderline QTc interval (QTc = 431–450 ms) but not of a prolonged QTc interval (QTc >450 ms)	(2) No female participants
	(3) Sample size: regular kratom users (*n* = 100) vs. non-drug-using control participants (*n* = 100)					(3) Participants were recruited from a single state in Peninsular Malaysia
						(4) Serum mitragynine analysis was not performed
						(5) Used Bazett’s formula to calculate QTc intervals

Note: hiPSC-CMs = human-induced pluripotent stem cell-derived cardiomyocytes, hERG = human ether-a-go-go-related gene, APD = action potential duration, IKr = rapid delayed rectifier potassium current, ICa,L = L-type calcium current, hERG1a/1b = the human ether-a-go-go-related gene 1a/1b current, HEK293 cells = hERG1a/1b-transfected human embryonic kidney 293 cells, Hsp90 = heat shock protein 90, , 7-HMG = 7-hydroxymitragynine, ECG = electrocardiogram, NPDS = National Poison Data System, RPC = Ramathibodi Poison Center, ROSC = return of spontaneous circulation, MRI = magnetic resonance imaging, and CT = computerized tomography.

## Results

### Kratom’s Adverse Cardiovascular Effects

A few studies have extracted data from the National Poison Data System (NPDS) in the United States and reported several adverse cardiovascular effects associated with kratom use. Indeed, most of the reported cases involved multiple exposures to various substances, including kratom, and only a minority of cases reported exposure to kratom only. Anwar et al. (2016) reported a total of 660 calls to the National Poison Data System (NPDS) in the United States from 2010 to 2015, showing an upward trend in kratom exposure from 26 calls in 2010 to 263 calls in 2015. Isolated kratom exposure was documented for 64.8% of these calls, and healthcare provider reports were documented for 75.2% of the calls. The most common cardiovascular symptoms and signs that these callers complained about were hypertension (11.7%) and tachycardia (25.0%) (Anwar et al., 2016).

Next, Post et al. (2019) examined 1,807 cases of kratom exposure in the United States that had been reported to the NPDS from 2011 to 2017. Again, this study indicated that kratom-related exposure cases were rising in the United States. Although 65.0% of these exposure cases were due to a single exposure to kratom, multiple-substance exposure was associated with more severe medical outcomes. The most common adverse cardiovascular effects and toxidrome reported in this study were tachycardia (21.4%), hypertension (10.1%), conduction defects (2.8%), chest pain (including non-cardiac pain; 2.6%), hypotension (1.8%), bradycardia (1.2%), and cardiac arrest (0.4%). However, this study was notably limited by examining unverified reports of kratom-related adverse effects and toxicity since these cases were self-reported and not confirmed by a poison control center (Post et al., 2019).

Davidson et al. (2021) retrospectively analyzed 938 cases of kratom exposure that had been reported to the NPDS in the United States (760 cases) or the Ramathibodi Poison Center (RPC) in Thailand (168 cases) from 2010 to 2017. This study found that co-exposure to kratom and other substances was more common in Thailand than in the United States (64.8 vs. 37.4%). Notably, this study revealed that tachycardia (30.4%) and hypertension (12.4%) were the most common adverse cardiovascular effects associated with kratom use (Davidson et al., 2021).

### Kratom’s Effects on Heart Rhythm and Cardiac Arrest Reports

Two *in vitro* studies, one cross-sectional study of human subjects, and a few separate case reports examined kratom’s effects on heart rhythm and cardiac arrest. The first study to identify evidence of kratom-related cardiotoxicity was an *in vitro* study which examined the effects of exposure of hERG-overexpressing human embryonic kidney (HEK) cells and hiPSC-CMs to mitragynine and its analogs (paynanthiene, speciogynine, and speciociliatine). The human ether-a-go-go-related gene (hERG) is a subunit of the potassium ion channel that regulates the rapid outward, delayed rectifier potassium current (I_Kr_) in the cardiomyocytes. Since cardiomyocytes from the human heart are not available due to safety concerns and technical shortcomings, the HEK cell presents a reliable alternative cell model to assess cardiotoxicity in *in vitro* studies. Meanwhile, hiPSC-CMs are generated from human-induced pluripotent stem cells via cardiomyogenic differentiation. Thus, hiPSC-CMs exhibit ionic current characteristics that resemble adult human cardiomyocytes. This *in vitro* study found that mitragynine at a concentration of 10 mM had suppressed the I_Kr_ in hERG-HEK cells. Meanwhile, mitragynine at IC_50,_ ranging from 0.91 to 2.47 mM, had also dose-dependently inhibited the I_Kr_ by 67–84% in hiPSC-CMs. Additionally, mitragynine had induced a marked hyperpolarization shift in the V_1/2_ of steady-state inactivation, in turn prolonging the action potential duration (APD) at 50 and 90% repolarization (439.0 ± 11.6 vs. 585.2 ± 45.5 ms and 536.0 ± 22.6 vs. 705.9 ± 46.1 ms, respectively). This finding indicated mitragynine’s potential to induce a prolonged QTc interval and increase the risk of torsades de pointes. However, mitragynine did not exhibit any tendency to suppress the voltage-gated calcium current (I_Ca,L_). Moreover, this study did not indicate that mitragynine could induce defects in hERG channel protein synthesis or the trafficking of ions, nor induce apoptosis of the hiPSC-CMs (Lu et al., 2014).

Next, a second *in vitro* study of kratom-related cardiotoxicity evaluated the mechanism of mitragynine-induced inhibition of the human ether-a-go-go-related gene 1a/1b (hERG1a/1b) current in stable hERG1a/1b-transfected human embryonic kidney (HEK) 293 cells. This study confirmed the previous findings by Lu et al. (2014) that mitragynine at an IC_50_ value of 332.70 nM had inhibited the hERG1a/1b current in a dose-dependent manner. Indeed, the IC_50_ value of mitragynine that had induced an inhibitory effect was lower than in the study by Lu et al. (2014). Additionally, this study also reported that mitragynine had decreased the fully glycosylated (fg) hERG1a protein expression at a lower concentration—but upregulated both core-glycosylated (cg) hERG1a protein expression and hERG1a-Hsp90 complexes at a higher concentration—after the hERG1a/1b-transfected HEK 293 cells had been exposed to mitragynine for 24 h. This finding highlighted the possibility that mitragynine could induce defects in channel trafficking of the hERG channel. The authors hypothesized that the upregulation of the hERG1a-Hsp90 complexes may be due to a mitragynine-induced hERG1a channel misfolding that activates the unfolded protein response (UPR) and endoplasmic-reticulum-associated protein degradation (ERAD) system (Tay et al., 2019). However, this possibility has yet to be investigated.

So far, only one study has evaluated electrocardiogram (ECG) findings related to regular kratom users (human subjects) without a history of polysubstance use or significant health problems (Leong Abdullah et al., 2021). This cross-sectional study compared ECG findings between regular kratom users who consumed kratom daily and a control group. The mitragynine concentration in the kratom juice consumed by the studied kratom users was also quantified and reported as a daily mitragynine intake of 434.28 mg. Several ECG abnormalities were documented among this study’s kratom users, such as sinus tachycardia (8% of all participants), left axis deviation (7%), prolonged QTc intervals (5%), a first-degree atrioventricular block (4%), left ventricular hypertrophy (4%), T inversion (4%), an incomplete right bundle branch block (3%), right axis deviation (2%), and sinus bradycardia (1%). The only ECG abnormality observed to be significantly prevalent among kratom users versus the control group was sinus tachycardia (OR = 8.61, 95% CI = 1.06–70.17, *p* = 0.035). Similarly, kratom users were also found to be more likely to experience borderline QTc intervals compared to the control group; however kratom users’ odds of prolonged QTc intervals did not increase versus the control group. Therefore, this study concluded that regular kratom consumption (at an average daily quantity of four glasses or with a mitragynine intake of 434.28 mg) can apparently increase QTc intervals but does not induce prolonged QTc intervals (Leong Abdullah et al., 2021). However, this study was limited in that it lacked serum mitragynine analysis. Therefore, further studies are needed to confirm these findings.

Despite a lack of human studies, a few case reports have pertained to kratom cardiotoxicity. Case 1 presented a 26-year-old man with no history of medical illness, who took no regular prescribed medication and who had visited an emergency department during cardiorespiratory arrest (primarily pulseless electrical activity). He had ingested an unknown quantity of kratom about 24 h prior to this incident. Upon examination, he was noted to have a brief period of ventricular arrhythmia. A computed tomography (CT) scan of the patient’s brain revealed imminent cerebral herniation, but a urine toxicology report indicated traces of codeine without the presence of other substances (a finding that was confirmed by the patient’s history revealing a standard dose of codeine prior to the incident). The patient died 12 h after an initial return of his spontaneous circulation, and his cause of death was suspected to be kratom-related cardiotoxicity. However, this report’s authors did not assess the patient’s serum mitragynine or 7-HMG levels. The quantity of kratom the patient had ingested prior to his death remained unknown (Aggarwal et al., 2018).

Case 2 presented a 35-year-old man with a significant past history of substance abuse. The patient had come under the care of emergency medical services (EMS) after suffering a cardiorespiratory arrest in his home. EMS and police personnel observed a large amount of kratom powder residue on the patient. Moreover, the patient had a history of alcohol, opioid, benzodiazepine, methamphetamine, and cannabis abuse. However, he had undergone rehabilitation treatment and, since then, abstained from all illicit drug use and alcohol. A systemic examination of the patient revealed no remarkable findings except for an examination of the central nervous system indicating marked reduced consciousness with a Glasgow coma scale of 3/15, as well as pinpoint, non-reactive pupils. A urine drug screen performed during the patient’s admission was negative for illicit drugs. Laboratory tests indicated hyperkalemia (potassium of 5.9 mmol/L), raised liver enzymes (aspartate transaminase of 282 IU/L and alanine transaminase of 273 IU/L), acidic blood with a significant anion gap, raised serum creatinine (3.0 mg/dl from a baseline level of 0.6 mg/dl), and high serum creatine kinase (4,000 U/L) and troponin I (0.37 μ/L). The patient’s other blood investigations were unremarkable. An echocardiography examination revealed cardiac arrest features while no other pathology was found. After treatment, the patient revealed a history of self-prescribed kratom consumption to treat his opioid dependence. He had consumed kratom multiple times daily to reduce his opioid withdrawal symptoms. In this case as well, however, the authors did not assess the patient’s serum mitragynine or 7-HMG levels. Moreover, the amount of kratom that the patient had ingested daily was not well quantified (Abdullah et al., 2019).

Case 3 described a 24-year-old man with a history of polysubstance abuse of an amphetamine-type stimulant, opioids, and benzodiazepine who had visited the hospital during a cardiorespiratory arrest. His history revealed no other risk of sudden cardiac death. This patient was unresponsive to multiple intravenous doses of naloxone. He experienced two episodes of polymorphic ventricular tachycardia for which defibrillation was performed. The first episode occurred while he was traveling to the hospital, and the second episode occurred during his initial admission to the emergency unit. A systemic examination of the patient’s cardiovascular system revealed no remarkable findings. The patient was placed on advanced cardiac life support, and his spontaneous circulation returned; however, his wide-complex tachycardia persisted. A urine drug screen was negative for opioids, cocaine, amphetamines, benzodiazepines, and tricyclic antidepressants. An investigation of the patient’s blood indicated hypokalemia (potassium of 2.9 mmol/L), while his other blood tests revealed circulatory arrest features. ECG findings reported an incomplete right bundle branch block while the patient’s echocardiogram was normal. After the patient recovered over 2 days, he described a history of continued polysubstance use, including kratom use. The amount of kratom he had consumed was not described, however, and the patient’s serum mitragynine and 7-HMG levels were not assessed (ElJack et al., 2020).

Case 4 described a 44-year-old man with a history of hypertension and hyperlipidemia on pharmacotherapy. He was physically active, performing routine daily exercise, and had obtained unremarkable results from an annual cardiac examination. This patient visited an emergency department due to multiple episodes of ventricular fibrillation, which required defibrillation. A family history revealed that the patient consumed a mixture of energy supplements containing kratom and caffeine (172–688 mg) daily. Laboratory blood investigations did not demonstrate any remarkable findings, but urine toxicology screening indicated the presence of ethanol. ECG findings indicated a prolonged QTc interval and an intraventricular conduction block, while a chest x-ray showed pulmonary vascular congestion. A further investigation with a CT scan of the brain, emergency cardiac catheterization, and cardiac magnetic resonance imaging (MRI) reported no abnormal findings (Sheikh et al., 2021).

### Kratom’s Association With Ischemic Heart Diseases and Other Cardiovascular Toxicities

Corkery et al. (2019) conducted a retrospective study that critically examined coroner and medical examiner reports, including autopsy reports of mortality cases associated with kratom use in the United Kingdom and beyond (including the United States, Germany, Canada, Ireland, Norway, Sweden, and Thailand) from 2008 to 2019. The authors successfully identified 156 deaths associated with kratom use. Only 16.7% of these mortalities were solely due to kratom exposure alone. The mean serum mitragynine level reported among the patients whose deaths were solely attributed to kratom use was 0.398 mg/L (range = 0.0035–0.890 mg/L; three cases). Meanwhile, the mean serum mitragynine level reported among the patients whose deaths had been associated with polysubstance use was 0.890 mg/L (range = 0.00089–16.000 mg/L; 62 cases). The mean serum level of 7-hydroxymitragynine among the patients whose deaths had involved polysubstance use was 0.662 mg/L (range = 0.0009–2.8 mg/L; five cases). Among the cardiovascular-system autopsy findings in cases linked to kratom exposure alone were coronary atherosclerosis (two cases), heart attack (one case), hypertensive cardiovascular disease (two cases), and left ventricular hypertrophy (three cases), totaling 5.1% of all studied mortality cases. Meanwhile, the autopsy findings linked to the co-administration of kratom with other substances included cardiac arrhythmia (one case), cardiomegaly (five cases), cardiomyopathy (one case), coronary atherosclerosis (five cases), focal band necrosis in the myocardium (one case), hypertensive cardiovascular disease (one case), left ventricular hypertrophy (three cases), and myocarditis (one case). However, this study’s main limitation was that it had collected data from a wide range of sources, and some of these sources’ quality was questionable (as data was extracted from case reports, coroner’s and autopsy reports, and data from special national mortality registry related to substance use), rather than data from more reliable studies, such as case control or cohort studies, or randomized controlled clinical trials. Therefore, the hierarchy of evidence that these data had contributed was not sufficiently reliable (Corkery et al., 2019).

## Discussion

Our literature review aimed to provide a comprehensive and timely description of kratom use’s adverse cardiovascular effects and cardiotoxicity risk. Based on our findings, we summarize a few salient features of the adverse cardiovascular effects and cardiotoxicity related to kratom use.

First, the most common acute adverse cardiovascular effects of kratom consumption were tachycardia and hypertension. Second, in the context of kratom’s effects on cardiac rhythm, a few *in vitro* studies reported that mitragynine—the most abundant psychoactive alkaloid in the kratom leaf—could induce prolonged QTc intervals and precipitate the risk of torsades de pointes in a dose-dependent manner. A few case reports also speculatively suggested that kratom consumption may have induced ventricular arrhythmia, particularly ventricular tachycardia and fibrillation, resulting in cardiopulmonary arrest. However, the findings of a recent study demonstrated that regular kratom consumption (the ingestion of a brewed kratom decoction) appeared to increase QTc intervals but did not induce a prolonged QTc interval or torsades de pointes (Leong Abdullah et al., 2021). Similarly, data from the national poison data system and autopsy reports of mortality cases indicated that conduction defects and cardiac arrhythmia were, indeed, rare.

Third, autopsy and coroner reports of deaths related to kratom use recorded a few cardiac pathologies related to myocardial ischemia, such as coronary atherosclerosis, focal band necrosis in the myocardium, and hypertensive cardiovascular disease. However, a study of ECG findings by Leong Abdullah et al. (2021) proved that myocardial ischemia (T-wave inversion) did not occur differently among kratom users versus the control group.

Fourth, concerning the risk of heart failure related to kratom use, autopsy and coroner reports of fatalities noted a few related cardiac pathologies, including left ventricular hypertrophy, cardiomegaly, and cardiomyopathy. Again, however, no significant differences were observed in the occurrence of left ventricular hypertrophy between kratom users and a control group (Leong Abdullah et al., 2021). Moreover, case reports did not indicate any features of heart failure related to kratom use.

Fifth, the risk of cardiotoxicity may increase with the co-administration of kratom alongside other substances. The mechanism underlying this finding may result from mitragynine’s role as a hepatic cytochrome P450 2D6 (CYP2D6) inhibitor that suppresses the metabolism of co-administered substances and increases their cardiotoxicity risk (Kong et al., 2011; Hanapi et al., 2013). Polymorphism of the CYP2D6 enzyme isoform categorized kratom users into a few sub-populations, such as ultra-rapid, extensive, intermediate, and poor metabolizers. Interestingly, co-administered substances that are also competitive CYP2D6 inhibitors of mitragynine could functionally convert kratom users who are extensive metabolizers to the poor metabolizers category *via* phenocopying (Bernard et al., 2006).

Finally, no animal studies have been conducted to investigate kratom’s effects on cardiovascular function. Animal studies are vital for assessment of toxicity related to a particular drug or compound. Animal studies allow the estimation of the lethal dose (LD_50_) related to cardiotoxicity of kratom or its pharmacoactive alkaloids, such as mitragynine or 7-HMG.

However, importantly, these findings should be interpreted with caution due to several limitations in these studies. First, human studies that have investigated the effects of kratom consumption on cardiac functioning and cardiotoxicity have been lacking—except for a cross-sectional study of ECG findings that was limited by its small sample size and lack of serum mitragynine concentration assessments among kratom users (Leong Abdullah et al., 2021). Furthermore, the findings of *in vitro* studies on cardiotoxicity should not be exclusively extrapolated to represent cardiotoxicity risk in humans. Second, despite a few case reports suggesting cardiotoxicity related to kratom use, the patients described in these case reports had either co-administered kratom with other substances (Aggarwal et al., 2018; Sheikh et al., 2021) or had a long, established history of polysubstance use that may have led them to co-administer kratom with other illicit substances (Abdullah et al., 2019; Eljack et al., 2020). Unfortunately, these case reports did not assess patients’ serum mitragynine levels. Third, although a few studies investigating national poisoning data, coroner reports, and autopsy reports suspected cardiotoxicity linked to multiple kratom-induced outcomes, a significant number of these cases had involved polysubstance use. Moreover, whether the described pathologies were caused by kratom use *per se* or had been partially compounded by underlying medical disorders is unclear. Another vital concern among kratom researchers pertains to the validity of published data since cases have been self-reported, without verification by a poison center, and these data’s hierarchy of evidence was not sufficiently reliable because most of these data had been obtained from case reports and descriptive studies (Corkery et al., 2019; Post et al., 2019; Davidson et al., 2021).

Despite these limitations, the data we examined in this literature review have allowed us to offer a few recommendations for future research. Despite the lack of related studies using a rigorous methodology, our findings suggest that chronic, regular kratom consumption may affect the cardiac rhythm and be associated with a risk of myocardial ischemia. Given the gap in the related research and kratom’s still unknown safety profile, more rigorous human studies with sufficiently large samples of respondents are urgently needed. Moreover, these studies should examine serum cardiac markers, echocardiograms, Holter monitoring, serum mitragynine levels, and serum 7-hydroxymitragynine levels in order to fully understand the potential cardiotoxicity risk of kratom use. Animal studies should, perhaps, also be conducted to determine the mechanisms underlying kratom use’s effects on cardiovascular function. Additionally, since *in vitro* studies have suggested that the upregulation of the hERG1a-Hsp90 complexes may be due to a mitragynine-induced hERG1a channel misfolding (Tay et al., 2019), a human study investigating whether kratom consumption activates the UPR and ERAD system would be interesting, potentially indicating kratom-induced endoplasmic reticulum stress. Finally, future case reports can be more informative than previous reports by including an assessment of serum mitragynine levels and, in the case of polysubstance use, the serum levels of other co-administered substances.

Thus, we cannot offer a definitive conclusion about kratom’s cardiotoxicity due to the lack of data and methodological limitations reported in existing studies. Nonetheless, our review offers two notable contributions to the literature. First, kratom’s most common adverse cardiovascular effects include tachycardia and hypertension. And second, kratom use may affect the cardiac rhythm in a dose-dependent manner. Therefore, a kratom overdose or the concurrent use of kratom with other illicit substances or medications that affect the cardiac rhythm (e.g., antiarrhythmics, antipsychotics, calcium channel blockers, beta-blockers, and antidepressants) may lead to cardiac arrhythmia. Moreover, the psychoactive alkaloids in kratom’s chemical profile remain poorly understood. Therefore, the question of whether kratom use can cause a cardiotoxicity risk merits further investigation.
